# Usability Evaluation of a Tablet-Based Intervention to Prevent Intradialytic Hypotension in Dialysis Patients During In-Clinic Dialysis: Mixed Methods Study

**DOI:** 10.2196/26012

**Published:** 2021-06-14

**Authors:** Matthew Willis, Leah Brand Hein, Zhaoxian Hu, Rajiv Saran, Marissa Argentina, Jennifer Bragg-Gresham, Sarah L Krein, Brenda Gillespie, Kai Zheng, Tiffany C Veinot

**Affiliations:** 1 School of Information University of Michigan Ann Arbor, MI United States; 2 School of Information and Computer Sciences University of California Irvine Irvine, CA United States; 3 Division of Nephrology Department of Medicine University of Michigan Ann Arbor, MI United States; 4 National Kidney Foundation New York City, NY United States; 5 Kidney Epidemiology and Cost Center University of Michigan Ann Arbor, MI United States; 6 Department of Internal Medicine University of Michigan Ann Arbor, MI United States; 7 Veterans Affairs Center for Clinical Management Research US Department of Veterans Affairs Ann Arbor, MI United States; 8 Department of Biostatistics, Consulting for Statistics, Computing and Analytics Research University of Michigan Ann Arbor, MI United States; 9 School of Public Health University of Michigan Ann Arbor, MI United States

**Keywords:** user interaction, dialysis, usability, informatics intervention

## Abstract

**Background:**

Patients on hemodialysis receive dialysis thrice weekly for about 4 hours per session. Intradialytic hypotension (IDH)—low blood pressure during hemodialysis—is a serious but common complication of hemodialysis. Although patients on dialysis already participate in their care, activating patients toward IDH prevention may reduce their risk of IDH. Interactive, technology-based interventions hold promise as a platform for patient activation. However, little is known about the usability challenges that patients undergoing hemodialysis may face when using tablet-based informatics interventions, especially while dialyzing.

**Objective:**

This study aims to test the usability of a patient-facing, tablet-based intervention that includes theory-informed educational modules and motivational interviewing–based mentoring from patient peers via videoconferencing.

**Methods:**

We conducted a cross-sectional, mixed methods usability evaluation of the tablet-based intervention by using think-aloud methods, field notes, and structured observations. These qualitative data were evaluated by trained researchers using a structured data collection instrument to capture objective observational data. We calculated descriptive statistics for the quantitative data and conducted inductive content analysis using the qualitative data.

**Results:**

Findings from 14 patients cluster around general constraints such as the use of one arm, dexterity issues, impaired vision, and lack of experience with touch screen devices. Our task-by-task usability results showed that specific sections with the greatest difficulty for users were logging into the intervention (difficulty score: 2.08), interacting with the quizzes (difficulty score: 1.92), goal setting (difficulty score: 2.28), and entering and exiting videoconference rooms (difficulty score: 2.07) that are used to engage with peers during motivational interviewing sessions.

**Conclusions:**

In this paper, we present implications for designing informatics interventions for patients on dialysis and detail resulting changes to be implemented in the next version of this intervention. We frame these implications first through the context of the role the patients’ physical body plays when interacting with the intervention and then through the digital considerations for software and interface interaction.

## Introduction

Chronic kidney disease (CKD) is the ninth leading cause of death in the United States [1,2]. The most advanced stage of CKD is end-stage renal disease (ESRD) wherein dialysis or transplantation is required for survival. In 2017, 746,557 Americans had ESRD [3]. However, transplants are not an option for many patients due to their health status and limited organ supply. Hemodialysis is the most common form of therapy for ESRD, with over 500,000 (about 70%) of all dialysis patients treated by hemodialysis rather than alternative dialysis modalities [3]. Hemodialysis is a demanding activity for patients, with a frequency of three times a week and each session lasting approximately 4 hours. The stability of these sessions varies, but an average of 20% of all hemodialysis sessions become unstable, most commonly due to intradialytic hypotension (IDH). Hemodialysis sessions are not always stopped early or interrupted. To improve the blood pressure, the patient is offered an intervention such as a bolus of saline or slowing of the ultrafiltration rate and/or placing the patient in the Trendelenburg position. IDH can result in cramping, dizziness, vomiting, fainting, and fatigue, with highly unstable sessions potentially leading to hospitalization or death [4-6]. Although IDH presents serious risks to patients undergoing hemodialysis, modifying patient behavior may prevent its occurrence. A promising approach to IDH prevention is to activate patients on hemodialysis to become more engaged in IDH prevention behaviors, such as monitoring their fluid intake and sodium consumption and ensuring they are dialyzed for their full prescribed times [7].

There is strong evidence suggesting that the use of digital informatics interventions is an effective way to support hemodialysis patient activation. In extensive reviews, Hibbard and Greene [8] and Sawesi et al [9] show that digital informatics interventions can enhance hemodialysis patient activation, health behaviors, and health outcomes. Hibbard and Greene [8] further conclude that activated patients on hemodialysis have better health outcomes and care experiences. Our previous work has shown that due to the fast pace of hemodialysis care in the United States, nursing staff are not able to perform additional tasks to educate patients in using new technologies or to perform troubleshooting activities [10]. Accordingly, we developed a digital informatics intervention to promote behavior change and activate patients on hemodialysis toward IDH risk reduction. Due to the lack of nursing staff time, we have also developed the intervention to include education of patients on hemodialysis to perform these tasks themselves. However, little prior work has considered how to design usable informatics interventions specifically for this patient population.

Similar work by Harrington et al [11] has investigated the use of a tablet-based application to support real-time monitoring and communication between patients and care providers. Their study looked at peritoneal dialysis at home, not in clinical settings, and was not developed with the aim of reducing a specific risk to which these patients are exposed. They also evaluated perceived satisfaction among the patients with using the application but did not examine interaction challenges. Furthermore, a systematic review of self-management interventions for patients with CKD identified a total of 23 studies that provide support for patients receiving dialysis [12]. These interventions were designed for specific tasks such as recording information (eg, meal logs and dietary intake), communicating with providers to monitor events and adherence to treatments, sending safety alerts for medications that may impair renal function, providing educational information, and monitoring of blood pressure and body weight. Another systematic review investigated different intervention types and evaluation models using mobile health technologies for the management of patients undergoing dialysis [13]. The systematic review found most functions of interventions to involve food tracking and self-monitoring. It also identified most outcome measures to be related to patient satisfaction or clinical effectiveness and did not evaluate interaction. Of the evidence provided by the two abovementioned systematic reviews [12,13], none of the studies reviewed had investigated the use of an eHealth intervention to improve hemodialysis patient outcomes for IDH or other challenges faced by these patients that can be addressed by self-management.

To develop informatics interventions for patients undergoing hemodialysis so that their risk of IDH is lowered, it is critical that they find the technology easy to use and that it provides a high-quality experience to them. We use the term “usability” to frame our investigation around the construct of interventions being easy to use, easy to learn, and easy to remember. Gould and Lewis [14] recommend three design principles when designing for usability. The first principle is that the system should have an early focus on the intended users and the tasks that they will need to accomplish by using the system. Accordingly, during early stages of development, we included users in the design process of this intervention [10,15-17]. Now that the first iteration of the previously developed intervention is available for patients on hemodialysis to interact with, this study aims to put into practice Gould and Lewis’ final two design principles: empirical measurement using qualitative or quantitative measures and iterative design process—learning from users and implementing that feedback into the next iteration of the design. In this study, we are particularly interested in the usability characteristics of effectiveness (are patients able to do learn what they need from the intervention?) and efficiency (can the patients easily learn and use the intervention on their own?). With this operationalization of usability, we aim to investigate how patients undergoing hemodialysis interact with the intervention and establish how to make the intervention usable for them when they run into difficulties [18].

Therefore, we pose the following research question to frame this study: What interaction challenges occur when patients undergoing hemodialysis use a tablet-based intervention in the dialysis clinic setting?

## Methods

### Description of the Intervention

Our previous work discusses the use of peer mentoring for young adults on hemodialysis [16,19], essential information design considerations of the hemodialysis clinical environment [20,21], information preferences of patients receiving dialysis [22], workflows used by clinicians to help prevent IDH [10], development of a patient-centered definition of unstable dialysis sessions [15], and results of a user-centered design process for developing the intervention evaluated in this study [17]. Based on this prior work, we have deployed a tablet-based intervention that was developed using the Ionic Framework’s app development platform along with AngularJS for the Android operating system, designed to run on a Samsung Galaxy tablet. The video player in the applications used the HTML5 video player Videogular2 for Angular.

**Figure 1:**
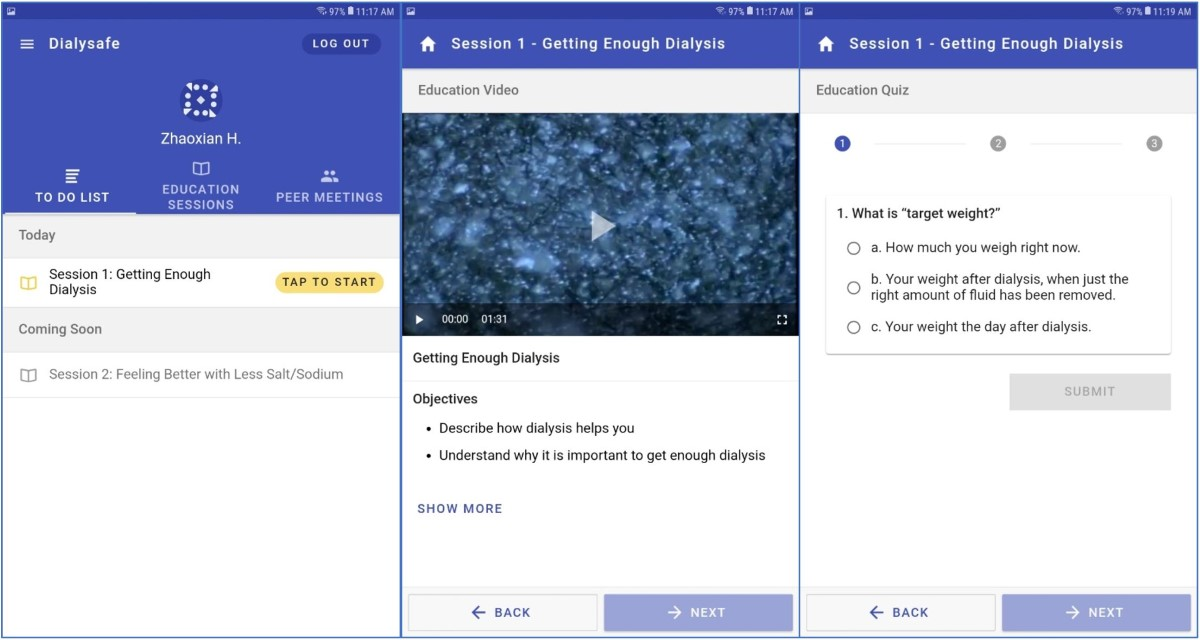
Screenshots from the tablet-based intervention: homepage showing the main to-do list (left panel), a typical educational model presenting content on intradialytic hypotension prevention with learning objectives (middle panel), and a quiz that is delivered after each session (right panel).

The intervention software (see screenshots in Figure 1) that patients interact with is organized into five sections, with each section covering one topic of IDH, that aim to activate patients on hemodialysis toward IDH prevention: (1) getting enough dialysis (which refers to completion of the amount of dialysis prescribed), (2) feeling better with less salt or sodium, (3) limiting fluid intake, (4) feeling better on dialysis and having easier sessions, and (5) getting more involved in one’s care. Each section includes educational content delivered via screencast, quizzes, story videos from other patients on hemodialysis, goal-setting modules, action plans, and motivational interviewing–based peer mentoring sessions delivered via tablet videoconferencing. All elements are to be delivered while the patients dialyze by using clinic-provided tablets, earphones, and Wi-Fi service. Clinic staff will provide tablets, disinfect them after each use, and place them in a charging cabinet.

After the patients undergoing hemodialysis log into the application by using their chosen username and password, the intervention presents a to-do list aligned with the current session theme. There are three tabs: The “To Do List” (Figure 1, left panel) acts as a homepage wherein the user can view all activities with which they can engage. Past educational content can be reviewed by navigating to the “Education Sessions” tab. Times and dates of future peer mentor sessions can be reviewed by navigating to “Peer Meetings.” Patients may view the themes of the upcoming sessions (see “Coming Soon”) on the homepage but may not be able to enter them until all prerequisite sessions are complete. It is important to note that peer mentor–mentee matching is managed by the National Kidney Foundation (NKF). Mentees undergo an intake procedure with the NKF staff to understand the patient’s characteristics, interests, illness experiences, and challenges. The NKF then uses this information to match compatible mentors to the mentee.

All five educational sessions begin with an educational video (Figure 1, middle panel). This is the main informative part of the session, featuring slides developed in collaboration with the NKF; a screencast video prerecorded by an NKF staff member that shows the slides and has a voiceover. Each video focuses on what patients on hemodialysis can do to lower their risk of IDH and the rationales for these actions. Session learning objectives, which are discussed in the first few minutes of each video, are listed below the embedded video player. To ensure that the patients receive the material, the video can only be skipped or fast-forwarded after the patient has viewed it once in full.

After viewing the video, patients are asked to test their knowledge on the content by answering a short quiz comprising two to four questions (Figure 1, right panel). All questions are multiple-choice questions, and answers refer to the session content.

**Figure 2:**
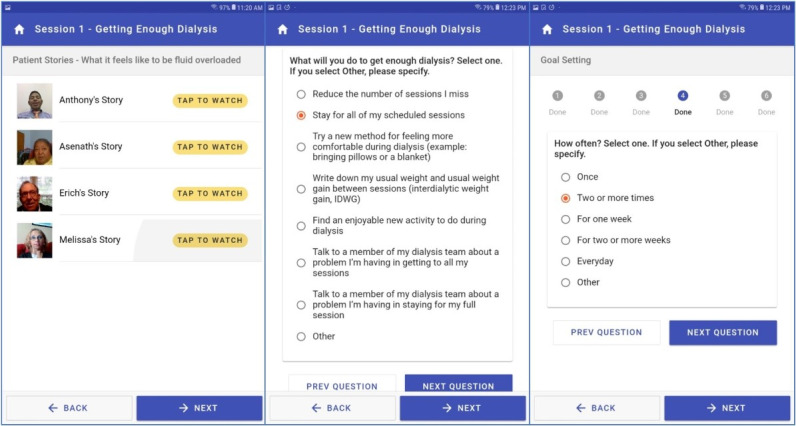
Screenshots from the tablet-based intervention showing a video library of hemodialysis patient stories (left), the quiz for module 1 (middle), and a question from the goal-setting module asking patients how often they will commit to adopting a certain behavior from the quiz.

The intervention includes a video library of hemodialysis patient stories (Figure 2, left panel). Each session includes two to four patient story videos in which real patients receiving dialysis share their answers to a question related to that session’s theme. All patients in the videos were recruited by the NKF. Patients who use the intervention may view as many patient story videos as they wish.

In the goal-setting module (Figure 2, middle and right panels), patients are prompted to select a goal that will help them improve their health behavior in relation to the theme. A set of prepopulated goals are provided, with content drawn from hemodialysis patient and care partner focus groups. Next, the patients select how often, how much, and when they will start the behavior (Figure 2, right panel). The options available on the goal-setting screen informs the motivational interviewing–based discussion between patients on hemodialysis and their peer mentors. This discussion is mediated by the application through a videoconferencing software that can also be accessed via the tablet. The answers provided by the patients on this screen are accessible to peer mentors through a peer mentor–facing web portal.

**Figure 3:**
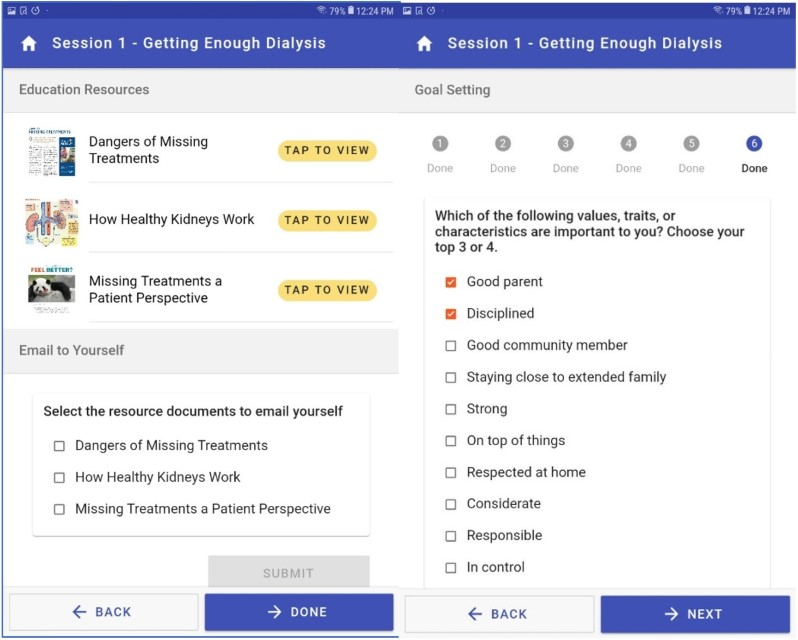
Screenshots from the tablet-based intervention: supplemental materials related to each learning module that patients can email themselves to review outside of dialysis hours (left panel); goal-setting module wherein patients select values, traits, or characteristics most important to them (right panel).

The intervention also provides the ability for patients on hemodialysis to obtain information when at the clinic or at home (Figure 3, left panel). Patients using the intervention can open and explore all documents listed within a session. They have the option of retaining the materials as “digital handouts” by sending a copy to themselves via email. This creates a simple process of collecting resources for review outside of dialysis hours.

In the first session, there is also a values selection exercise in which patients can choose from a list or write their own list of values, traits, or characteristics most important to them (Figure 3, right panel). Examples of values mentioned in the intervention are “being a good parent,” “being a good spouse or partner,” “being competent,” or “feeling energetic.” This element of the intervention is informed by prior work that shows linking values to health goals is a powerful tool to support behavior change [23]. This effectively motivated the patients’ health behavior goals with their “why” for making a change (ie, *why* they choose to take action to prevent IDH)—because of the values that they select in the exercise. These values are viewable by the peer mentor and form the basis of discussion with patients about their own personal reasons for effecting a change.

### Recruitment

Prior work on sample size for usability studies has shown that, for the size and complexity of our mobile app, a total of 15 users is acceptable to discover virtually all software problems [24-26]. We recruited 14 patients over a 2-day period at a University Hospital hemodialysis clinic in a Midwestern State, which has about 83 patients. The facility models team-based care where teams are composed of nurses, patient care technicians, a dietitian, social worker, physicians, and advanced practice providers. Patients receiving hemodialysis that were awake while dialyzing were approached by the clinical staff. Clinicians informed these patients about the study and asked if they were interested in participation. Two research staff members then approached interested patients to obtain informed consent. To ensure that the intervention was appropriately designed for its most likely users, patients were intentionally selected to be representative of the demographics of patients on hemodialysis in terms of race, age, and gender [27]. All interviews and observations were conducted in the dialysis clinic setting. The usability testing was cross-sectional, and participants only had access to the intervention during the user testing. The study was declared exempt from oversight by the Institutional Review Board (HUM00159531) at the University of Michigan.

### Data Collection

Data were collected using two methods to leverage the (1) the think-aloud method [28,29] and (2) structured researcher observation of patients on hemodialysis using the intervention. We used a think-aloud–based interview guide containing the questions for tasks to be completed on each screen of the application; this method permitted the collection of participants’ subjective perceptions of usability challenges. We also used a data collection instrument to capture objective observational data regarding what interaction tasks the patients had attempted and a score for how difficult each task was for them. Two field researchers were trained to take notes and assign scores. All data gathered were based on objective criteria for which available data were highly visible. These criteria include participants’ difficulty with performing each task: (1) on their own with little difficulty, (2) with some difficulty, or (3) with considerable difficulty and requiring assistance to complete the task. We applied these scores for tasks such as logging into to the application, reviewing the “to-do” list, playing and stopping a video, taking a quiz, and entering and exiting a video conference meeting with a peer mentor. As patients worked their way through the tasks, they were asked to verbalize their thoughts, explaining how they interpreted the screens, what they thought they would experience at each stage, or what questions or problems they had. While patients were describing their thoughts, a researcher took field notes, including direct quotes. Each observation session lasted from 20 minutes to 1 hour. The evaluation session began when field researchers sat with a patient to have them navigate through the application to perform each task.

### Data Analysis

We entered the numerical scores (on the scale of 1 to 3) for each task level of difficulty into a spreadsheet and calculated mean scores for each interface task in a Microsoft Excel spreadsheet. We also created a case-by-case display to capture field notes [30,31] alongside each patient’s interaction difficulty score to evaluate reasons for the difficulty scores. Finally, we performed qualitative content analysis to inductively categorize field notes and organize them into themes concerning challenges experienced by patients on hemodialysis in using the intervention software.

## Results

### Participant Demographics

Table 1 below shows the gender, race, age range, and previous touch screen experience of the study participants.

**Table 1:** Study demographics of patients undergoing hemodialysis (N=14) and their experience in using touch screen devices.

Characteristic		Participants
**Gender, n (%)**		
	Male	7 (50)
	Female	7 (50)
**Race, n (%)**		
	White	5 (35.7)
	African American	9 (64.3)
**Age range (years), median (IQR)**		
	41-74	58 (12.5)
**Touch screen experience, n (%)**		
	Yes	9 (54.3)
	No	5 (35.7)

### General Constraints

Observations showed that patients undergoing hemodialysis have *limited mobility and reach* depending on where their access port for dialysis needles is located on their arm or chest. Because chest access confers an elevated risk of serious infections, many patients have access in their arms. With an arm access, patients have large needles inserted into their arm, appended with soft tubes through which blood is removed and then returned. This connection to the machine is sensitive to movement, typically leading to limited mobility in their arm. Consequently, as one patient noted, “In dialysis, I only have one hand to use.” This limits how patients can hold and tap the tablet. If they used a thumb to tap the screen because of these issues, the device often failed to register their tap. Patients are further constrained during blood pressure checks—as exclaimed by one patient during usability tests, “The machine's got me tied up now.” In such situations, patients required help to navigate the tablet. The inability of the patient to operate the tablet also influenced the usability data we were able to collect. At times, when the field researcher would ask a participant to select a specific menu item or play a video, the participant could not physically perform the task at that moment. This led to difficulty in using the intervention because it left the patient in a position where they could not be able to do what they wanted to do, when they wanted to do it.

Our observations also revealed that *dexterity issues* are common in patients undergoing hemodialysis. For instance, those who experienced limited dexterity or tremors while dialyzing faced a challenge in being able to accurately tap in the intended area. This created a feedback deficiency—patients did not know when they had activated the button after having tapped on it several times. Aware of this difficulty, one patient suggested, “You may want to add clicks or noises when typing,” to help patients know when they touched the right spot. This was not an issue of learning; they did not need to know how to use the device but rather wanted to know if their input was accepted by the device, if they pressed it correctly, or if they touched the screen in the right manner in the right location.

Impaired vision and use of reading glasses are also common in patients on hemodialysis, due in part to diabetes and hypertension being the most common causes of kidney failure. Accordingly, the intervention was designed to accommodate a general need for large print and buttons, as well high-contrast text and images. Despite designer efforts, there were legibility issues with reading a smaller font on one of the screens; during the think-aloud sessions with the quiz feature, several patients stated, “I can't read it,” while taking the quiz.

Furthermore, we noted a difference between participants’ perceptions based on their previous experiences with touch interfaces. About two-thirds of our sample (9/14, 64%) had prior experience with touch screens, whereas the remaining one-third did not have any touch screen experience. Patients without touch screen experience were confused when the screen timed out when they were not interacting with the tablet, and they did not intuitively know how to tap elements on the screen, sometimes holding taps for too long or too short a time or too lightly. In some cases, the patients did not have prior knowledge of interaction through tapping the screen, or of what elements would not respond to a tap. The clearest example of this was patients not knowing how to interact with the slider on the quiz screen, as described below.

### Task-by-Task Usability Results

The usability evaluation revealed several aspects of the intervention application with which patients had difficulty interacting. Some of these difficulties were not limited to the intervention application but extended to the accessibility of, and configuration options for, the Android mobile operating system and video player running underneath the intervention application. As described below, many of the interaction challenges observed concerned visibility and readability of text and problems with gestures or taps.

**Figure 4:**
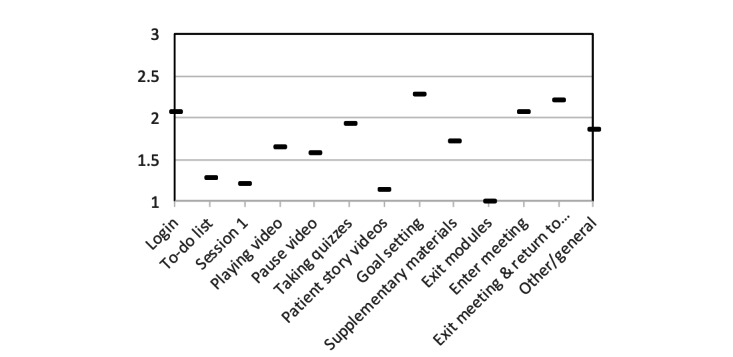
Difficulty of use scores (scale of 1 to 3) for each intervention application feature. A score of 1 indicates the participant was able to accomplish the task on their own with little difficulty. A score of 2 indicates some difficulty and a score of 3 indicates considerable difficulty requiring assistance to complete the task. The y-axis represents the difficulty score, and the x-axis shows the task performed by the patients.

Figure 4 shows the average usability scores for each assigned task based on ratings—using a scale of 1 to 3—of how difficult the tasks were for patients. The easiest interactions were watching videos and exiting learning modules. The most challenging tasks, which often required help to complete, were goal setting, exiting live video chats, and returning to the intervention application from the video chat application. We also identified several usability issues that were pervasive throughout interactions with the intervention but became more pronounced when using certain screens. These pervasive issues included tapping, reading text, selecting text entry fields, and registering interactions when touching the screen. Each of these issues are explored further below.

The login screen had an average score of 2.07. What patients found challenging here was selecting the appropriate text entry field; furthermore, half of the patients had problems with the keyboard layout. Specifically, they struggled to find the back key, had trouble reading or interpreting legends on the keyboard, confused a zero (0) with the letter “O,” had problems with case sensitivity and accidently engaging caps lock, or were unfamiliar with typing an email address on a touchscreen keyboard and voice input functionality displayed on the keyboard. The keyboard was available as is in the Android operating system and was not specifically designed for patients on hemodialysis or for the study intervention.

Once logged in, the patients were greeted with the to-do list. Although this screen was unique to the intervention, it was easy for them to navigate, interact with, and understand; the mean difficulty score was only 1.28 (SD 0.73). Some reports concerned needing larger print for certain items, additional formatting—such as bold typeface and larger font—to help with clarity of understanding list items, and minor visual design enhancements that would help the patients understand the purpose of the to-do list.

Patients tested the first session of the intervention. They had little difficulty with this activity (difficulty score: 1.21). However, playing videos for the first learning session posed slightly more difficulty as this section received a score of 1.64 and saw multiple patients struggling with controlling the audio volume level and the screen timing out. We also asked the participating patients to pause the video (difficulty score: 1.57); several patients did not know how to do this and needed a prompt, as well as instructions as to how to exit the video when it was set to full screen.

The intervention quizzes required patients to make dexterous tap gestures to select and submit responses to multiple-choice questions. This proved to be a difficult task (difficulty score: 1.92) for the patients on hemodialysis due to the use of only one hand, and even more so for those who had tremors, unsteady hands, or lotion or other fluid applied on their hands. This complicated the ability of a capacitive touch screen to register touch gestures; one patient summarized this experience in the following colorful manner: “This thing is a pain in the ass [sic]” (stated twice), and then, “This thing is really...you have to tap it light.” It is important to note that this was the first screen in the intervention that challenged some patients’ understanding of interactivity, as some patients would tap too long or too quickly or tried to double tap certain parts of the screen to select answers. This screen also presented a challenge in interpretation, with one patient reporting, “I don't get this, where to put my answer,” and then, “I’m not sure where to get the answer” about how to interact with the quiz.

Patients were asked to view patient stories, pause the video, exit the full-screen video, and navigate back to the video to continue watching. Patients faced little resistance in performing these tasks; this had an average difficulty score of 1.14. While reviewing this material, some patients also gave feedback that they enjoyed the videos and saw them as beneficial; for example, one participant said, “It's some interesting stuff on here if you are new to dialysis…If you are new and it's your first time on dialysis, this is good.”

For all but 3 participants (11/14, 78%), goal setting proved to be the most challenging and difficult intervention screen for patient interaction (difficulty score: 2.28), requiring guidance to complete all interaction tasks. Specifically, the slider for some of the quiz questions proved to be difficult to interact with; for instance, one patient said, “That's a tricky button.” Additionally, there were also *Next* and *Previous* question buttons just above the *Back* and *Next* buttons for the main application. This was a frequent point of confusion for patients; one said it was “A little confusing, time consuming.” Another patient asked, “Is this different NEXT?...the extra step probably would get tedious for some people.” Lastly, this task showcased some of the touch interface problems that were symptomatic of observed challenges in the use of a touch screen device. One patient commented, “It looked like it went somewhere, but it didn't“ (referring to multiple touches to get the screen to register). Another voiced a problem with predicting how the screen would react to touch: “[I] shouldn't touch anything before I finish reading.”

As stated, the intervention allows patients to email a PDF file of supplementary materials to their personal email address. Patients found a specific issue with the placement of the *Next* button on the screen such that if they double tapped the screen, they could miss the prompt to email the document to themselves. Patients required relatively little prompting (difficulty score: 1.71) for this screen. After these tasks, the patients were directed to exit the learning module, a step that was completed with no challenges or need for support; it was the only task that with a difficulty score rating of 1.0.

When using this intervention, patients will need to interact with a video conferencing application to connect with peer mentors; thus, the application is programmed to automatically launch a meeting in the videoconferencing platform BlueJeans [32]. Patients had moderate difficulty (difficulty score: 2.07) with this task. Most observed challenges concerned how to join video meetings, and there were interface issues in the external video conferencing software. For example, to join a meeting room in the video software, patients needed to press a white *Join* button; however, almost half of the patients (6/14, 43%) thought it was a notification or text, and not a button. Accordingly, they were not aware that they needed to press it. Other parts of the video interface also became confusing, with one patient saying aloud, “I'm looking at controls at bottom, because it is red, not sure if I should hit it.” Patients had similar, moderate challenges of navigating the interface to exit the video chat room and returning to the main intervention application (difficulty score: 2.21).

## Discussion

### Principal Findings

The intervention was developed from in-clinic observations, interviews, and patient focus groups that included participatory design activities. Issues related to impaired vision and dexterity were raised in this early work and were taken into account during the design of the intervention. Nevertheless, we found that despite our efforts in this area, the intervention needed further refinement to address these issues.

Mobility challenges unique to patients undergoing dialysis concern restrictions to movement owing to the use of devices such as blood pressure machines, dialysis machines, associated wires and tubes, as well as tight clinical spaces or dialysis chairs [21]. These patients need the ability to comfortably hold and manipulate a tablet while sitting on the dialysis chair so that they can use it during hemodialysis. For the intervention to be successful and provide a high-quality experience, patients should not have to wait to engage with the intervention until they can fully operate the tablet with both hands (due to blood pressure check-ups or other examinations). Physical limitations need to be accommodated through an interface that can move, adapt, and be responsive. Technologies such as eye tracking could be used, along with thumb-based interaction, to better support body orientation and gaze of patients on hemodialysis with mobility issues.

Another movement-related challenge for these patients was dexterity; this included the use of hands and fingers to hold the tablet and interact with the interface. Aging-related differences are observed, ranging from decrease in grip strength and pinch strength to deterioration of nerve receptors [33]. Typical dexterity issues in aging hands can be exacerbated by vascular and cardiac problems commonly observed in patients on hemodialysis. This creates problems for touch interface–based interactions. Problems include not receiving feedback on interface elements such as buttons, keyboards, scroll bars, and text entry fields. It is difficult for patients receiving dialysis to navigate the interface of the intervention and receive no acknowledgement of what interface element is interactive or whether the system has registered their input. Audio feedback either through spoken word or tones would provide better support for patients, which can be coupled with haptic feedback to provide additional physical response and visual cues that indicate interface input. However, visual cues need further consideration as detailed below.

Like touch, vision changes with age, requiring designers to attend to the specific needs of users to deliver a more equitable interaction experience [34]. Vision challenges in our patient sample include easily identifying text, controls, buttons, icons, and other parts of the interface. As previously mentioned, although visual cues can be utilized to convey interface interaction, it is important that these be consistent and standardized. To accomplish this, there is a need to leverage design patterns for presenting and perceiving information such as differentiating between interface elements and visual notifications [35].

Without addressing these concerns related to the physical body and accessibility of patients on hemodialysis, informatics interventions risk introducing unwanted challenges to the participants, potentially resulting in disengagement with, and abandonment of, the intervention—a particularly common result in digital informatics interventions with marginalized groups [36]. Indeed, health status and age are correlated with less use of health technologies [37]. We argue that design should be used to advocate for patients receiving hemodialysis that are often marginalized with respect to how they can use technology and how technology meets their needs to challenge existing disparities in health technology uptake, usage, and benefits. Thus, we stress that patients receiving hemodialysis have a right to amelioration of such interaction problems to create equal opportunities to benefit from health technologies [38].

### Limitations

Our study has several limitations. One potential limitation is that we did not use a validated usability instrument such as the Health Information Technology Usability Evaluation Scale (Health-ITUES) or System Usability Scale. However, this was not done because the primary goals of the study were to identify what usability challenges patients on hemodialysis face and how they can be addressed. Another limitation of the intervention may be that the difficulty scores reported in our study were rated by the field researcher and not the participant. We chose this method to obtain objective data and because the field researcher was familiar with the software and trained in using the scale we derived, thus allowing for more accurate measurement of whether tasks were completed. However, these observations were complemented by use of the think-aloud protocol, through which a research team member recorded participants’ verbal accounts of their thoughts while they completed the assigned tasks, thus leading to rich accounts of the types of difficulties that they faced. Nevertheless, future usability evaluations might leave an opportunity for developing a self-report scale for patients undergoing hemodialysis to quantitatively rate their perceived difficulty in completing tasks using the intervention. A further limitation is that, due to the presence of researchers associated with the intervention, participants may have been less critical of the intervention than they might otherwise been. However, we note that the study revealed several usability issues in need of correction. Another limitation of this study is that patients were not selected based on whether they have a shunt or fistula providing dialysis access and how different forms of dialysis access might influence mobility of their dominant hand when using the intervention**.**

Finally, based on our findings, we developed a list of changes to be implemented in the next version of the intervention; some of these are described below. We increased the font size where needed to make the pages more readable, adjusted the button and button row margin to make it easier to tap, and increased the font size in the alert dialog box. On the goal-setting form, we ascribe both “save” and “next” functions to a singular “save button => next button”. The objective with these changes was to reduce the number of required clicks, as we observed that extensive tapping was a problem for patients receiving hemodialysis, particularly those using one hand or their thumbs. Furthermore, on the goal-setting form, we changed the slider bar to the toggle button group. Observations of patient interaction revealed difficulty using one of the sliders to select an option rather than tapping; thus, we changed it to a group of buttons, and each button represented a value in the range. On both the goal-setting form and the quiz screen, we removed the footer section (“Back” and “Next” buttons) and changed the “Next” button for the last question function such that it transitions to the next section. This streamlined interaction for patients on hemodialysis and further reduced the number of taps they needed to make. We also changed values to columns rather than a long page; this reduced the need for scrolling, which was important, because the need for scrolling indicated missed content, as some patients were not aware that they needed to scroll down the page. We also designed a patient training module on both the use of touch screen devices and the intervention itself.

### Conclusions

This study evaluated the usability of a digital intervention to engage patients on dialysis that is intended to reduce the patient’s risk of IDH when dialyzing. A task-by-task analysis of each screen of the intervention identified usability challenges related to setting goals with a high difficulty score of 2.28, and interacting with the videoconferencing platform, which had a high difficulty score of 2.07 to join a meeting and a score of 2.21 to leave the videoconferencing session and return to the main intervention application. Furthermore, our analysis revealed four general constraints when designing for dialysis patients: dexterity in touch and interface navigation, limitations in movement and device positioning when dialyzing, readability and vision challenges for older patients due to small-sized text, and a disparity between patients on hemodialysis who had no experience with tablets and those who had literacy and knowledge of mobile tablet use. These constraints and challenges in user interaction can prevent or defer effective use of the intervention. When developing informatics interventions for patients on hemodialysis, it is critical that such usability challenges are prevented and that the technology’s affordances are leveraged to do so. Future design of informatics interventions for patients receiving hemodialysis should proactively account for these usability issues so that they may achieve their intended effects.

## Abbreviations

CKD: chronic kidney disease
ESRD: end-stage renal disease
Health-ITUES: Health Information Technology Usability Evaluation Scale
IDH: intradialytic hypotension
NKF: National Kidney Foundation
PCORI: Patient-Centered Outcomes Research Institute
